# Inactivated Nevus Tissue with High Hydrostatic Pressure Treatment Used as a Dermal Substitute after a 28-Day Cryopreservation Period

**DOI:** 10.1155/2021/3485189

**Published:** 2021-02-24

**Authors:** Yoshitaka Matsuura, Michiharu Sakamoto, Shuichi Ogino, Jun Arata, Naoki Morimoto

**Affiliations:** ^1^Department of Plastic and Reconstructive Surgery, Otsu Red Cross Hospital, Shiga 520-8511, Japan; ^2^Department of Plastic and Reconstructive Surgery, Graduate School of Medicine, Kyoto University, Kyoto 606-8507, Japan; ^3^Department of Plastic and Reconstructive Surgery, Graduate School of Medicine, Shiga University, Shiga 520-2192, Japan; ^4^Department of Plastic and Reconstructive Surgery, National Hospital Organization Kyoto Medical Center, Kyoto 612-8555, Japan

## Abstract

**Background:**

Giant congenital melanocytic nevi (GCMN) treatment remains controversial. While surgical resection is the best option for complete removal, skin shortage to reconstruct the skin defect remains an issue. We report a novel treatment using a high hydrostatic pressurization (HHP) technique and a cryopreservation procedure. However, cryopreservation may inhibit revascularization of implanted nevus tissue and cultured epidermal autograft (CEA) take. We aimed to investigate the influence of the cryopreservation procedure on the HHP-treated dermis specimen and CEA take on cryopreserved tissue.

**Methods:**

Nevus tissue harvested from a patient with GCMN was inactivated with HHP of 200 MPa and then cryopreserved at -30°C for 28 days. The cryopreserved specimen was compared with fresh (HHP-treated without cryopreservation) tissue and with untreated (without HHP treatment) tissue to evaluate the extracellular matrix, basal membranes, and capillaries. Cultured epidermis (CE) take on the cryopreserved tissue was evaluated following implantation of the cryopreserved nevus tissue with CE into the subcutis of nude mice.

**Results:**

No difference was observed between cryopreserved and fresh tissue in terms of collagen or elastic fibers, dermal capillaries, or basement membranes at the epidermal-dermal junction. In 4 of 6 samples (67%), applied CE took on the nevus tissues and regenerated the epidermis in the cryopreserved group compared with 5 of 6 samples (83%) in the fresh group.

**Conclusion:**

Cryopreservation at -30°C for 28 days did not result in significant damage to inactivated nevus tissue, and applied CE on the cryopreserved nevus tissues took and regenerated the epidermis. Inactivated nevus tissue with HHP can be used as a dermal substitute after 28-day cryopreservation.

## 1. Introduction

Giant congenital melanocytic nevi (GCMN) are large, brown-to-black skin lesions present at birth. GCMN are >20 cm in diameter, comprise an area >100 cm^2^, or involve >2% of the total body surface area [1–5], and are associated with a risk of malignant transformation to melanoma [2–6]. The frequency of malignant transformation has been reported to range from 0.7% to 15.4% [1–3]; therefore, treatment should be considered for two reasons: to reduce the chances of malignant transformation and for cosmetic reasons. However, the treatment of GCMN remains both challenging and controversial, and a variety of surgical approaches such as excision, dermabrasion [7, 8], curettage [9–11], chemical peels, cryotherapy, and laser treatment [12, 13] have been performed. Among these, surgical resection is the only method for complete GCMN removal and should be first considered to prevent the emergence of melanoma; however, skin shortage to reconstruct the skin defect remains a major challenge.

To overcome skin shortage postcomplete resection, we previously reported a novel treatment strategy for GCMN using a high hydrostatic pressurization (HHP) technique. HHP at 200 MPa inactivates all kinds of human skin cells, such as keratinocytes, fibroblasts, endothelial cells, and nevus cells [14–16]. In our novel therapy, resected nevus tissue is inactivated through HHP and is then reused and applied to the skin defect at the original site for dermal reconstruction. A cultured epidermal autograft (CEA) is then applied on the inactivated nevus for epidermal reconstruction approximately 3 weeks later. We undertook a clinical trial to elucidate the feasibility and efficacy of this protocol [16]. Our results showed that the inactivated nevus tissue was revascularized at the implanted site and that CEA could take and regenerate the epidermis on the revascularized nevus tissue.

In the current protocol, the inactivated nevus tissue was returned to the skin defect immediately after the HHP procedure during the operation, as this preservation method involving inactivated nevus tissue had not been investigated. We, then, aspired to preserve the inactivated nevus tissue for several weeks for subsequent use as a dermal substitute in the subsequent operation. Preservation of the inactivated nevus tissue would provide us an optimal duration of time for preparing the wound bed prior to implantation.

Cryopreservation seems to be the most suitable means to preserve inactivated nevus tissue, however, it has been reported that cryopreservation could damage cells and proteins [17–20] and may inhibit the revascularization of implanted nevus tissue and CEA take on the nevus tissue. Therefore, the influence of the cryopreservation procedure on the dermis and the CEA take ratio should be investigated. In this study, we cryopreserved inactivated nevus tissue for 28 days and evaluated basal membranes, capillaries, and extracellular matrix (ECM) structures. Moreover, we implanted the cryopreserved nevus tissue with cultured epidermis (CE) in the subcutis of nude mice and evaluated the CE take.

## 2. Materials and Methods

This study was approved by the Ethics Committee of Kyoto University Graduate School and Faculty of Medicine (permit no. R1263). Regarding animal research, our experiment was performed in compliance with standard guidelines concerning animal experiments at Kyoto University and was approved by the Animal Research Committee of Kyoto University Graduate School of Medicine (permit no. Med Kyo 18107).

### 2.1. Nevus Tissue Preparation

**Figure 1:**
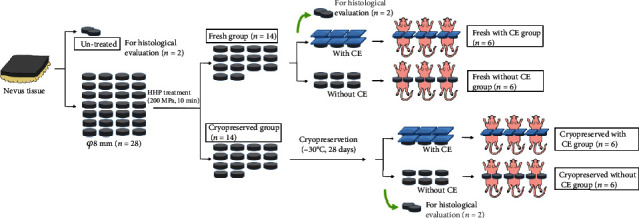
Scheme of experiment flow. CE: cultured epidermis; HHP: high hydrostatic pressure.

Nevus tissue was obtained from a male patient who had a GCMN covering a large area of his head and neck who had undergone surgical resection of the nevus lesion on the left side of the neck. In total, 40 cm^2^ of the resected nevus specimen was used in this study after having obtained written patient consent. The specimen was stored at 4°C in a refrigerator until the next step. We prepared 30 round pieces of nevus tissue (diameter, *φ*8 mm) from the resected nevus tissue using a disposable skin biopsy punch (TOREPAN® (Kai industries co., Ltd, Japan)), and subcutaneous adipose tissues were removed with scissors. Six of the prepared *φ*8 mm nevus tissue samples were used for histological or electron microscopic survey as untreated nevus tissue. Twenty-four samples were used for the next step after allocation into two groups: (i) a “fresh” group, where nevus tissues were evaluated and implanted into nude mice with/without CE immediately after HHP and (ii) a “cryopreservation” group, where nevus tissues were evaluated and implanted into nude mice with/without CE after cryopreservation for 28 days following HHP (Figure 1).

### 2.2. Pressurization of the Nevus and Cryopreservation after Pressurization

Pressurization was performed using a previously reported portable HHP device [21]. This system consists of a main pressure unit with a hydraulic hand pump, an electric pressure generation unit, and a pressure control unit. During the HHP process, a pressure-tight cell was pressed with a piston via an electric compressor or hand pump. Pressure up to 280 MPa can be applied using this device. Before pressurization, the nevus tissues in the fresh group were soaked in normal saline solution (Otsuka Pharmaceutical Co., Ltd., Tokyo, Japan) and in a 10% glycerol solution (Glyceol, Chugai Pharmaceutical Co., Ltd., Tokyo, Japan) in the cryopreservation group, using sterile plastic bags. The samples in both groups were pressurized at 200 MPa for 10 minutes at room temperature. After pressurization, the samples in the cryopreservation group were preserved at -30°C for 28 days. They were then rapidly thawed in a warm bath at 37°C immediately prior to use.

### 2.3. Evaluation of Dermal Structures and Basement Membranes in the Cryopreserved Nevus Tissues

A total of six samples (untreated nevus tissue [*n* = 2], fresh group [*n* = 2], and cryopreserved group [*n* = 2]) were used for histological or electron microscopic survey to evaluate their dermal structure and basement membranes.

One sample in each group was fixed in 10% neutral buffered formalin solution (FUJIFILM Wako Pure Chemical Co., Ltd., Osaka, Japan) at room temperature overnight and then embedded in paraffin wax. Axial sections from the center of the samples were prepared and stained with hematoxylin and eosin (H&E), azocarmine-aniline blue (AZAN), Elastica van Gieson (EVG), or immunochemical staining with collagen type IV or von Willebrand factor (vWF). AZAN staining was used to evaluate collagen fibers in the dermis, and EVG staining was used to distinguish elastic fibers from collagen fibers. Immunochemical staining with collagen type IV and vWF was used to identify the basement membranes at the epidermal-dermal junction and vascular endothelial cells, respectively.

For the electronic microscopy survey, the other samples in each group were fixed in 0.1 M phosphate buffer with 2% glutaraldehyde and 4% paraformaldehyde at 4°C overnight. After postfixation with 1% osmium tetroxide for 2 hours, they were dehydrated and dried. For scanning electron microscopy, the samples were coated with a thin layer of platinum palladium. The specimens were examined using an Hitachi S-4700 scanning electron microscope (Hitachi Co., Ltd., Tokyo, Japan) to investigate the precise structure of the collagen fibers in the dermis. For transmission electron microscopy, thin sections were stained with saturated uranyl acetate and lead citrate and were observed using an Hitachi H-7650 electron microscope (Hitachi Co., Ltd., Tokyo, Japan) to investigate basement membranes at the epidermal-dermal junctions.

### 2.4. Immunochemical Staining

For immunochemical staining, paraffin-embedded sections were deparaffinized and rehydrated and subsequently incubated in antigen retrieval solution (Proteinase K (Dako Japan Co., Ltd., Tokyo, Japan)) at room temperature. Anti-vWF rabbit polyclonal antibodies (dilution 1 : 5,000, code A0082 Dako Japan Co., Ltd., Tokyo, Japan) or anticollagen type IV gout polyclonal antibody (dilution 1 : 15000 code 1340-01; SBA, Birmingham, USA) was used as the primary antibody. Histofine® Simple StainTM Mouse MAX-PO (Nichirei Biosciences Inc., Tokyo, Japan) was then applied as the secondary antibody and exposed to 3-3′-diaminobenzidine-4HCl (DAB, Nichirei Biosciences Inc.), and counterstaining was performed using hematoxylin.

### 2.5. Implantation of the Pressurized Nevus with CE into the Subcutis of Nude Mice

#### 2.5.1. CE Preparation

Human CE was prepared by the Japan Tissue Engineering Co., Ltd. using Green's method described previously [22, 23] with some modifications. Briefly, cryopreserved keratinocytes cultured from human skin obtained from a supernumerary finger resected in an operation of a polydactyly patient were thawed and disseminated on irradiated 3T3-J2 cells used as a feeder layer. Keratinocytes were cultured in Dulbecco's Modified Eagle Medium and Ham's F12 medium (mixed 3 : 1) and supplemented with 5% fetal calf serum, insulin, hydrocortisone, cholera toxin, triiodothyronine, epidermal growth factor, and antibiotics in an atmosphere of 10% CO_2_ at 37°C. Human CE was obtained as keratinocyte sheets, which were detached from flasks after treatment with dispase and then backed with a nonwoven fabric sheet of 10 × 8 cm in size. The CE sheets were cut into 1 cm square sheets before use.

#### 2.5.2. Implantation and Evaluation of Epidermal Regeneration

Twelve nude 6-week-old male mice (BALB/c-nu, Japan Charles River Co., Ltd, Tokyo, Japan) were acclimatized for one week before treatment. They had *ad libitum* access to regular sterile food and water. They were allocated into four groups (three mice in each group): “fresh with CE,” “fresh without CE,” “cryopreserved with CE,” and “cryopreserved without CE.” All painful treatments were performed under general anesthesia with inhalation of isoflurane (Pfizer, Co., Ltd, Tokyo, Japan). Isoflurane was administered at 5% for induction and at 1–1.5% for maintenance. The dorsum of each mouse was incised, and two pockets in the subcutaneous layer were created on both sides of the dorsum, and one sample was implanted in each pocket (two samples in each mouse). Prior to implantation, inactivated epidermis from all the pressurized nevus samples was removed using forceps (the epidermis is invariably damaged due to pressurization at 200 MPa and the damaged epidermis can be easily removed through scratching or pinching). Following this, in the “fresh with CE” group, immediately pressurized (200 MPa) nevus tissue was implanted with CE onto the surface of the subcutaneous pockets of the nude mice. In the “fresh without CE” group, pressurized nevus tissue was implanted without CE. Similarly, in the “cryopreserved with CE” group, nevus tissue that had been cryopreserved at -30°C for 28 days after 200 MPa pressurization was implanted with CE. In the “cryopreserved without CE” group, cryopreserved nevus tissue was implanted without CE. The dorsum was then sutured using 5-0 nylon sutures (Diadem; Medical U&A, Inc., Osaka, Japan). All the mice were euthanized with exposure to carbon dioxide gas 14 days after implantation, and the implanted nevus specimens were harvested. Photographs of the nevus specimens were taken, and the specimens were fixed with 10% neutral buffered formalin solution, embedded in paraffin blocks, and cut into 5 *μ*m-thick sections. The sections were subjected to H&E staining.

Regeneration of the epidermis on the pressurized nevus tissue was evaluated under optical microscopy (Biorevo BZ-9000; Keyence, Co., Osaka, Japan).

## 3. Results

### 3.1. Histological Evaluation of the Cryopreserved Nevus Tissue

**Figure 2:**
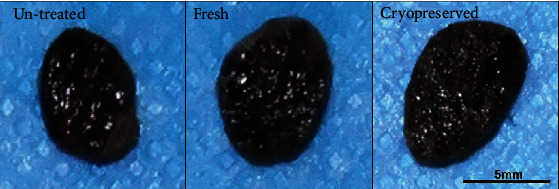
Nevus tissues resected from a patient with giant congenital melanocytic nevi. Untreated, nevus tissue without HHP treatment. Fresh, nevus tissue treated with HPP at 200 MPa. Cryopreserved, nevus tissue cryopreserved at -30°C for 28 days after HHP treatment at 200 MPa. No morphological differences such as shape, color, or size are observed. HHP: high hydrostatic pressurization.

**Figure 3:**
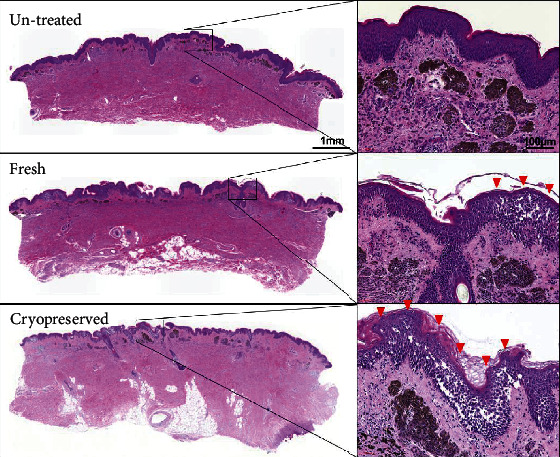
H&E-stained sections of nevus tissue in untreated, fresh, and cryopreserved groups. Massive vacuolation was observed (red arrowheads) in the epidermal layer in the fresh and cryopreserved group. Nevus cell nests existed in the dermal layer and melanin pigmentation still remained in all the groups.

The nevus tissues in the untreated, fresh, and cryopreserved groups are shown in Figure 2. No morphological differences in terms of shape, color, or size were observed among the three groups: the pigmentation of the nevus tissues had not decreased with HHP treatment or cryopreservation. The elasticity of the nevus tissues was similar among the three groups. H&E-stained sections are shown in Figure 3. Massive vacuolation was observed in the epidermal layer in the fresh and cryopreserved groups (HHP-treated groups), which indicated that keratinocytes had been injured and inactivated due to the HHP treatment. In contrast, the epidermal component remained intact in the untreated nevus samples. Otherwise, no obvious change in the dermal layer was observed. Nevus cell nests were observed in the dermal layer, and melanin pigmentation had remained.

**Figure 4:**
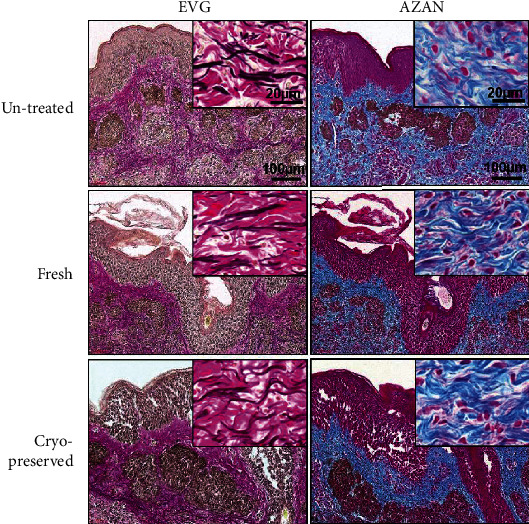
Sections stained with EVG or AZAN. Elastic fibers (stained brown in EVG) and collagen fibers (stained pink in EVG and blue in AZAN) showed no obvious change from untreated tissues in both the fresh and the cryopreserved groups. AZAN: azocarmine and aniline blue; EVG: Elastica van Gieson.

The damage to elastic and collagen fibers was assessed using EVG- or AZAN-stained sections (Figure 4). Elastic fibers were stained black with good differentiation from collagen fibers and could be clearly visualized in EVG staining. The morphologic characteristics of elastic fibers in the fresh and cryopreserved groups showed no obvious changes from those in the untreated tissues, with the thickness of the elastic fibers and their distribution among the collagen fibers being similar, and with no fragmentation of fibers observed.

Collagen fibers were stained blue in the AZAN-stained sections. The collagen fibers observed in the three groups (untreated, fresh, and cryopreserved) had similar thicknesses and densities, and no collagen fiber fragmentation or swelling was observed.

### 3.2. Immunohistochemical Staining of Basement Membranes and Blood Vessels

**Figure 5:**
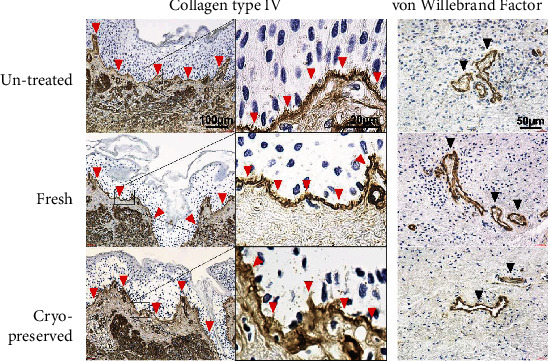
Left: immunochemically stained sections with collagen type IV. The basement membrane, indicated with red arrowheads, has absolute consistency and a distinct structure in all the groups. Right: immunochemically stained sections with von Willebrand factor (vWF). Black arrowheads indicate the capillaries. The structure of the vessels remained intact in all the groups.

The immunohistochemically stained sections with anticollagen type IV antibody or anti-vWF antibody are shown in Figure 5. Type-IV collagen is a component of the basement membrane; therefore, the basement membrane is stained brown. The epidermis was vacuolized, and keratinocytes were dispersed due to the HHP treatment in the fresh and cryopreserved groups (the hollow in the epidermal layer in the untreated group was an artifact). However, the basement membrane had absolute consistency and distinct structures in all groups, which indicated that neither the HHP treatment nor cryopreservation had damaged the basement membrane.

The endothelial cells had reacted with the anti-vWF antibody, and the blood vessels located in the dermal layer were stained brown. The morphological structure of the vessels and the consistency of the tunica intima remained intact in both the fresh and cryopreserved groups, which indicated that HHP treatment and cryopreservation had not damaged the structure of the blood vessels.

### 3.3. Electron Microscopic Survey for Collagen Fibers and Basement Membranes

**Figure 6:**
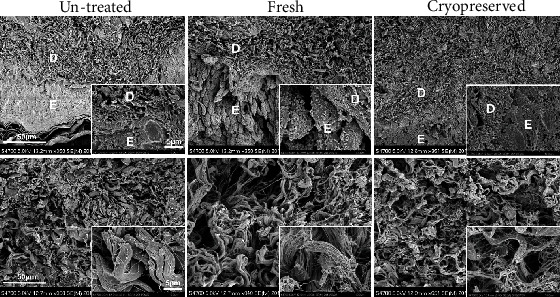
Scanning electron microscopy images of untreated, fresh, and cryopreserved nevus tissues. The epidermis disappeared in the fresh and cryopreserved groups. Densely packed collagen fibers constitute the dermal layer with no vesiculation or laceration, and the density of the collagen fibers appeared to be almost the same in all groups. No fibril fragmentation or swelling was observed among the three groups. D: dermis; E: epidermis.

Figure 6 shows the scanning electron microscopy images of the nevus tissues in the untreated, fresh, and cryopreserved groups. The epidermis had detached from the dermis or disappeared in the fresh and cryopreserved groups. Densely packed collagen fibers constituted the dermis layer with no vesiculation or laceration, and the density of the collagen fibers appeared to be almost the same in all groups. Fine collagen fibrils had gathered together to form large fibers, and no fragmentation or swelling of fibrils was observed.

**Figure 7:**
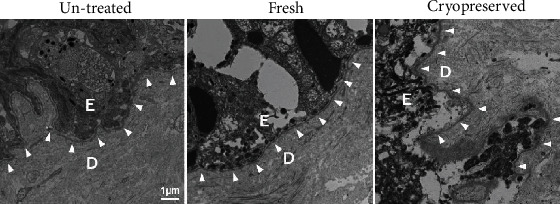
Transmission electron microscopy images of untreated, fresh, and cryopreserved nevus tissues. The arrowheads indicate the basement membranes. The basal layer of the epidermis is rigidly connected to the surface of dermis in the untreated group, while keratinocytes are destroyed and fragmented into small pieces in the fresh and cryopreserved groups. The lamina densa in the basement membrane is clearly observed among the three groups. D: dermis; E: epidermis.

Figure 7 shows transmission electron microscopy images of the epidermal-dermal junction of the nevus tissues in the untreated, fresh, and cryopreserved groups. In the untreated nevus, the basal layer of the epidermis was tightly connected to the surface of the dermis at the level of the basement membrane and the lamina densa can be clearly identified. In contrast, in the fresh and cryopreserved groups, keratinocytes were destroyed and fragmented into small pieces and had become detached from the dermis. However, the lamina densa in the basement membrane was clearly observed without any fragmentation or disruption.

### 3.4. Epidermal Regeneration of Cryopreserved Nevus Tissue after CE Application

**Figure 8:**
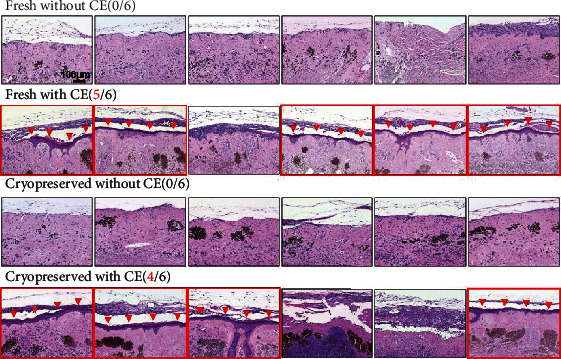
H&E-stained sections of nevus tissues harvested 14 days after implantation in nude mice. Red arrowheads indicate regenerated epidermis. No epidermal regeneration was observed in all six samples in both the fresh without CE and cryopreserved without CE groups. Epidermal regeneration was observed in five of six samples in the fresh with CE group, and in four of six samples in the cryopreserved with CE group (the images of the samples with epidermal generation are surrounded with a red line). CE: cultured epidermis.

Epidermal regeneration was evaluated with the samples harvested 14 days after implantation with or without CE application (Figure 8). First, we confirmed that the epidermis would not regenerate after implantation without CE application because the keratinocytes of the nevus tissues had been totally inactivated due to the HHP treatment. No epidermal regeneration was observed in all six samples in both the fresh without CE group and the cryopreserved without CE group. This result ensured that the epidermis observed in the HHP-treated nevus tissues after implantation with CE would be derived from the applied CE sheets and not from the epidermal stem cells of the nevus tissues. Finally, epidermal regeneration was observed in five of six samples in the fresh with CE group and in four of six samples in the cryopreserved with CE group. Nevertheless, not all the samples achieved epidermal regeneration, and our findings indicated that CE was able to take onto cryopreserved nevus tissue.

## 4. Discussion

In this study, our results showed that CE took on inactivated nevus tissue cryopreserved at -30°C for 28 days. CE is a cultured keratinocyte sheet manufactured from a patient's own skin biopsy and has been found to be effective in the treatment of severe burns [24–26]. However, when CE is applied onto a wound surface without a dermal component such as exposed deep fascia or adipose tissue, the take ratio of CE is unsatisfactory [27]. Therefore, reconstruction of the dermal component is necessary prior to CE application. In burn wound treatment, cadaver skin transplantation and/or combination therapy with a widely expanded skin autograft are used to reconstruct the dermal component [26, 28]. For GCMN treatment, we developed a new treatment for dermal reconstruction using an HHP procedure, and a clinical trial for GCMN was performed that combined treatment using an HHP procedure and CE [16].

HHP processing is a widely used method for preserving and sterilizing food, in which a product is processed under very high pressure, leading to the inactivation of certain microorganisms and enzymes in the food. In previous reports, we elucidated that all cells contained in the skin were inactivated with no damage to the dermal component, including the basal membrane, at 200 MPa of HHP treatment [15, 29], which indicated that inactivated nevus tissues might be a useful dermal component for cutaneous reconstruction.

Cryopreservation is generally used to store living cells, tissues, or chemicals. However, the cryopreservation procedure can damage proteins and cells due to vitrification, cold shock, osmotic injury, and intracellular ice formation [30, 31]. Cryopreserved skin tissue has been reported to damage cells and reduce viability after thawing compared with fresh skin tissue [18, 19]. Therefore, it is possible that cryopreservation of inactivated nevus tissue may damage the basement membrane at the epidermal-dermal junction; damage the extracellular matrix, affecting collagen and elastic fibers, and/or damage capillaries in the dermis; therefore, inhibiting revascularization of inactivated nevus tissue after implantation and/or CE take on them. In this study, the influence of the cryopreservation procedure was evaluated through comparing cryopreserved nevus tissue at -30°C for 28 days with fresh (noncryopreserved) tissue. With cryopreservation, glycerol was used as a cryoprotectant to reduce cryo-injury, in accordance with our previous study [32]. No difference was observed in relation to collagen fibers, elastic fibers, or to capillaries in the dermis or the basement membrane in the epidermal-dermal junction between the cryopreserved tissue and fresh tissue. This finding indicated that cryopreservation at -30°C for 28 days did not result in significant damage to inactivated nevus tissue. We then evaluated the CE take on the cryopreserved nevus tissue in a nude mouse model. In 4 of 6 (67%) samples, CE took on the nevus tissues and regenerated the epidermis in the cryopreserved group, compared with 5 of 6 (83%) samples in the fresh group, which indicated that CE is able to take on the cryopreserved nevus tissue and to regenerate the epidermis as cryopreservation did not cause critical damage to the nevus tissue. Because of the small sample size, it was not possible to discuss the statistically significant difference in the take ratio between the cryopreservation and fresh groups.

Our study findings indicated that inactivated nevus tissue can be kept for 28 days prior to its implantation. This extended waiting period afforded us the time to prepare the wound bed prior to inactivated nevus implantation to improve revascularization. An increased blood supply from the wound bed, as a result of wound bed preparation such as negative pressure wound therapy, has been shown to improve the success rate of a skin graft [21]. Therefore, the take ratio of the implanted nevus tissue may be improved due to wound bed preparation prior to implantation. We suggest that dermal regeneration with much thicker nevus tissue would likely improve the cosmetic result, thus, reducing hypertrophic scar formation and/or pigmentation. Moreover, resected nevus tissues that are discarded during conventional treatment can be stored and reused as a dermal component in a subsequent operation.

One limitation of this study concerned the discrepancy in the implantation procedure. More specifically, inactivated nevus tissues were implanted into the subcutis of nude mice, whereas these tissues would be implanted on an open wound surface such as exposed adipose tissue in a clinical setting. Therefore, further studies are needed to confirm whether cryopreserved inactivated nevus tissues would be revascularized after implantation and whether CE application would be accepted when used in a clinical setting. Furthermore, we did not undertake a long-term observation in this study. The influence of cryopreservation on scar formation should also be evaluated, and we intend to undertake further studies to address these issues.

## 5. Conclusion

We showed that the extracellular matrix, capillaries, and basement membranes of nevus tissues inactivated using the HHP procedure preserved their integrity after cryopreservation at -30°C for 28 days. Moreover, CE applied to the cryopreserved nevus tissues took and regenerated the epidermis. Cryopreservation of inactivated nevus tissues provided sufficient time to prepare the wound bed prior to implantation, and GCMN treatment with HHP improved the patient's outcome.

## Data Availability

All data of the study is included in the manuscript.
